# Structure formats of randomised controlled trial abstracts: a cross-sectional analysis of their current usage and association with methodology reporting

**DOI:** 10.1186/s12874-017-0469-3

**Published:** 2018-01-10

**Authors:** Fang Hua, Tanya Walsh, Anne-Marie Glenny, Helen Worthington

**Affiliations:** 1 Department of Orthodontics, Hubei-MOST KLOS & KLOBM, School and Hospital of Stomatology, Wuhan University, Luoyu Road No.237, Wuhan, 430079 China; 2 Cochrane Oral Health, Division of Dentistry, School of Medical Sciences, Faculty of Biology, Medicine and Health, The University of Manchester, Manchester Academic Health Science Centre, Oxford Road, Manchester, M13 9PL UK

**Keywords:** Reporting quality, Structured abstracts, Randomized controlled trials, Consort, Editorial policies, Publishing

## Abstract

**Background:**

The reporting of randomised controlled trial (RCT) abstracts is of vital importance. The primary objective of this study was to investigate the association between structure format and RCT abstracts’ quality of methodology reporting, informed by the current requirement and usage of structure formats by leading general medical/internal medicine journals (secondary objective).

**Methods:**

A two-part cross-sectional study. First, through hand searches, we identified all RCTs published in the top-50 high-impact general medical/internal medicine journals during July–December 2015 (*n* = 370), and retrieved the ‘instructions to authors’ of these journals. From these, we extracted the actual usage of structure formats and headings, as well as relevant journal policies. Then, after a pilot study and sample size calculation, we assessed the methodology reporting quality of 176 IMRaD (Introduction, Methods, Results, and Discussion) and 165 HS (Highly Structured) RCT abstracts sampled from 33 of the 50 selected journals, using a 9-item checklist developed based on the CONSORT for Abstracts guidelines (primary outcome: overall quality score, OQS; score range 0 to 9).

**Results:**

88% (324/370) of all identified RCT abstracts were structured, among which 66% (215/324) used the IMRaD format and 34% (109/324) used HS. According to journals’ ‘instructions to authors’, 48% (24/50) journals required IMRaD, 32% (16/50) required HS, 8% (4/50) required unstructured, while the rest did not state any requirement on structure format. According to generalised estimation equation analysis adjusting for potential confounders and clustering effects, the OQS of HS abstracts was 0.5 (95% CI 0.1 to 1.0, *p* = 0.028) higher than IMRaD abstracts. More HS abstracts reported study setting (adjusted odds ratio, 4.2; 95% CI: 1.7 to 10.0; *p* = 0.001), definition of the main outcome measure (2.5; 1.3 to 4.9; *p* = 0.006) and the time point for main outcome assessment (3.0; 1.5 to 6.2; *p* = 0.002), whereas more IMRaD abstracts described the unit of randomisation (0.4; 0.3 to 0.8; *p* = 0.004).

**Conclusions:**

For RCT abstracts, the IMRaD format is more frequently used and required by leading general medical/internal medicine journals than the HS format. Abstracts in the HS format report trial methodology more completely than those in the IMRaD format.

**Electronic supplementary material:**

The online version of this article (10.1186/s12874-017-0469-3) contains supplementary material, which is available to authorized users.

## Background

Abstracts are the first and often the only part of a medical research report that is read [1–3]. Many healthcare professionals base their initial assessment of a study or even clinical decision-making on abstracts alone [4–6]; many journal editors screen research papers by reading only the abstracts [7]. Therefore, complete, transparent and accurate reporting of abstracts is vital. Thirty years ago (1987), Dr. Haynes and colleagues [8, 9] first proposed the use of structured abstracts for clinical study reports, to help readers identify and appraise articles more quickly, help authors summarise their studies more explicitly, facilitate peer review, and allow more precise electronic literature searches [9]. Soon after, structured abstracts were adopted by major medical journals (e.g. *Annals of Internal Medicine*, *British Medical Journal*, *Journal of the American Medical Association*) [10–13] and then became more and more widely used in the medical literature [14, 15].

Many previous studies have suggested that structured abstracts are more informative than unstructured abstracts [16–18]. However, not all structured abstracts of medical research articles followed the original ‘8-heading’ format proposed by Haynes et al. [9] (*Objective*, *Design*, *Setting*, *Patients or participants*, *Interventions*, *Main outcome measures*, *Results*, *Conclusions*) [14, 19]. In fact, according to Nakayama et al. [19], among structured abstracts published in the top-30 general medical/internal medicine journals in 2001, only one-third used the 8-heading format (or its variations), while the other two-thirds were in ‘IMRaD’ (***I****ntroduction*, ***M****ethods*, ***R****esults*, **a**nd ***D****iscussion*), a simpler format that was first adopted by the *New England Journal of Medicine* in 1990 [20].

The main difference between IMRaD and highly structured (HS) formats (e.g. the original 8-heading format) is that the *Methods* heading of IMRaD is usually split into multiple, more specific headings (e.g. *Design*, *Setting*, *Participants*, *Interventions*, *Main outcome measures*) in HS formats [19]. This could mean that HS formats are more effective than IMRaD in reminding authors to report key details about their study methodology. However, whether and to what extent such effect exists remains unclear, because to our knowledge there has been no study directly comparing the reporting quality of structured abstracts in different formats (e.g. IMRaD vs. HS).

In evidence-based medicine, at the level of primary research, high-quality randomised controlled trials (RCTs) are considered the highest-level evidence for determination of the benefits and harms of health care interventions [21, 22]. To standardise and improve the reporting of RCT abstracts, the CONsolidated Standards Of Reporting Trials (CONSORT) group released the CONSORT for Abstracts guidelines in 2008 [23]. Thereafter, many studies have used these guidelines to assess the reporting quality of RCT abstracts published in medical journals [24–37], several of which compared the reporting quality of structured and unstructured abstracts [24–28]. However, results of these studies have been inconsistent, and again, none of them has made a direct comparison between IMRaD and HS abstracts.

Therefore, we carried out this study primarily to investigate the association between structure format and methodology reporting quality of RCT abstracts, with a null hypothesis that there is no difference in the overall quality of trial methodology reporting in HS abstracts and IMRaD abstracts. Additionally, as there has been no recent update of Nakayama et al.’s [19] 2005 findings on structure format usage, our secondary objective was to present the current requirements and actual usage of different structure formats by leading general medical/internal medicine journals for RCT abstracts.

## Methods

This cross-sectional study was written in accordance with the STROBE guidelines for reporting observational research [38, 39]. A copy of the protocol of this study is available in Additional file 1.

### Definitions of structure formats

Based on relevant definitions used by the U.S. National Library of Medicine [40] and other researchers [19, 41], we categorised a priori common abstract structures into three formats:

- Unstructured format: abstracts presented in one paragraph, with no distinct, labelled sections;
- IMRaD format: structured abstracts with four distinct main sections labelled with *Introduction*/*Background*/*Objective(s)*, (*Materials*/*Patients* and) *Methods*, *Results*, and *Discussion*/*Conclusion(s)* respectively, with or without other separate sections for trial registration and/or source of funding;
- HS format: structured abstracts with more than four distinct, labelled main sections and at least one of the five headings (*Design*, *Setting*, *Patients*/*Participants*, *Interventions*, *Main outcome measures*) that Haynes et al. [9] proposed for methodology reporting, with or without other separate sections for trial registration and/or source of funding.

### Journal selection

The Thomson Reuters 2014 Journal Citation Report (JCR) [42] was used to select fifty journals that were listed under the ‘Medicine, General & Internal’ category, had the highest impact factors and publish RCTs. During the selection process (in April 2016), the ‘instructions to authors’ of all potentially eligible journals were retrieved from their official websites and examined. We excluded journals that 1) were inactive/discontinued; 2) do not publish primary research/RCTs; or 3) only publish solicited research, according to their journal instructions and websites.

### Part 1: Structure format usage and journal policies

#### RCT identification

The official online archives of all fifty selected journals were hand-searched to identify RCT reports published in these journals during July–December 2015. Pre-determined inclusion criteria for RCTs included: human participants, experimental design, comparative/controlled trial, healthcare-related interventions, as well as random allocation to interventions [27, 43]. When the eligibility of a study could not be determined based on its title and abstract, the corresponding full-text was retrieved and screened. One author (F.H.) carried out the hand-search and screening, and reviewed the screening results with other authors.

#### Data extraction

From the ‘instruction to authors’ of each selected journal, we extracted journal type (general/specialty), word limit for abstracts, structure format required, headings required, whether detailed instruction was given for each heading required, and the endorsement level of the CONSORT for Abstracts reporting guidelines. In accordance with previous similar studies [44–46], endorsement level was categorised into:

- Not mentioned: the reporting guideline (RG) was not mentioned in the instruction;
- Recommended: the instruction suggested that the RG ought to be considered or used (e.g., ‘should…’, ‘please…’, ‘we suggest/encourage authors to…’);
- Required: the instruction stated that adherence to the RG is a condition for publication, or the corresponding RG checklist was required to be submitted (e.g., ‘authors must…’, ‘authors are required to…’).

For each identified RCT abstract, we extracted the following information: title of article, title of journal, structure format, headings used, overall word count, word count for Methods section (or its equivalent part), overall number of paragraphs, number of methodology paragraphs, overall number of heading terms, number of heading terms regarding methodology, and whether all the eight headings proposed by Haynes et al. [9] were used. One author (F.H.) carried out the data extraction and examined all extracted data with other authors.

### Part 2: Structure format and methodology reporting quality

#### Pilot study

An internal pilot study was performed to calibrate authors in assessment of reporting quality, to indicate necessary refinement of the assessment protocol, and to inform sample size calculation.

Firstly, we excluded all journals with no RCT identified in Part 1 of this study, due to the expected difficulty to include enough RCTs from these journals and their unknown actual usage of structure formats. Secondly, we categorised all remaining journals into three groups (unstructured, IMRaD, HS) according to the structure format that they only or mainly used during July–December 2015. Journals that published an equal number of RCT abstracts in different formats were grouped according to the format that they required in their journal instructions. Then, using a stratified random sampling method (with each journal as a stratum), we chose 12 unstructured abstracts from unstructured journals, 12 IMRaD abstracts from IMRaD journals, and 12 HS abstracts from HS journals [47].

All authors (F.H., T.W., A.-M.G., H.W.) assessed these 36 abstracts independently and in duplicate, using a 9-item checklist based on the CONSORT for Abstracts guidelines and relevant explanations [23], for the assessment of methodology reporting quality of RCT abstracts (Table 1; Additional file 1). For each quality item, we gave a score of ‘1’ when the item was adequately reported, and a score of ‘0’ when the reporting was inadequate. Then for each abstract, we calculated an overall quality score (OQS; range, 0 to 9), the primary outcome of this study, by totalling the scores for all nine items [24, 27].

**Table 1:** The checklist used for assessment of methodology reporting in this study, modified from the CONSORT for Abstracts guidelines

Items/Supplementary items^a^	Criteria/Content^b^
1. Design	Explicit description of the trial design (e.g. parallel, cluster, crossover)
2. Participants	Eligibility criteria for participants
3. Setting	Settings where the data were collected
4. Interventions	Interventions intended for each group
5. Outcome	Clearly defined primary/main outcome(s) for the trial
*5a. Time point*	When was the primary/main outcome(s) assessed
*5b. No. of outcomes* ^c^	The number of described primary/main outcome(s)
6. Random assignment	Clear statement that participants were allocated to groups in a randomised manner
*6a. Unit of randomisation*	Description of the unit of randomisation (e.g. patients, schools, communities)
7. Sequence generation	Method used for random sequence generation
8. Allocation concealment	Method used for allocation concealment
9. Blinding (Masking)	Whether or not participants, caregivers, and those assessing the outcomes were blinded to group assignment
*9a. Generic blinding*	Generic description only (e.g. single-blind, double-blind)

CONSORT for Abstracts: the CONSORT (Consolidated Standards of Reporting Trials) extension guidelines for reporting of RCT abstracts [23]

^a^Only the scores of main quality items (no.1 to 9) contributed to the primary outcome (overall quality score, range: 0 to 9); supplementary items (5a, 5b, 6a, 9a) were documented for information purposes only

^b^Detailed scoring criteria are available in the online Appendix

^c^A continuous variable, not dichotomous

After all authors had completed their assessments, meetings were held for discussions regarding inter-examiner discrepancies and possible refinement of the scoring criteria. A unanimous set of assessment results for the 36 pilot abstracts was reached and used for sample size calculation. First, we calculated a minimum sample size based on the mean OQS and standard deviation (SD) of each group, and an assumed smallest effect of interest of 0.5 in OQS. We then inflated the minimum size by 8% (non-central t-distribution approach; for pilot study sized 12 per arm, type I error 5%, power 80%) to account for the fact that a sample estimate of the variance, rather than the ‘population’ variance, was used in the calculation [48].

Since the mean OQS (SD) derived from our pilot study was 2.67 (0.78) for the unstructured group, 4.00 (1.54) for IMRaD, and 5.08 (1.00) for HS, based on the IMRaD group SD and the assumed minimum effect of interest, a sample size of 162 abstracts was required for each group according to the above-mentioned calculation method.

#### Full study

##### Sample creation

As the number of journals varied among journal groups (unstructured, IMRaD, HS), for each group we designated a separate minimum number (*n*) of RCT abstracts to include from each journal, so that the calculated sample size could be obtained. Then, for instance, when an IMRaD journal published more than *n*_*i*_ (*i* for IMRaD format) IMRaD RCT abstracts during July–December 2015, we used an online random number generator (Research Randomizer; www.randomizer.org) to randomly choose *n*_*i*_ IMRaD abstracts to include for that journal. In contrast, when an IMRaD journal published less than *n*_*i*_ IMRaD RCT abstracts during the period, we carried out a PubMed search (search term, “*the journal title*”[Journal] AND “randomized controlled trial”[Publication Type]) to retrieve recent RCT abstracts published in the same journal before July 2015, until *n*_*i*_ abstracts were included for that journal. Before inclusion, all RCTs identified through electronic searches were examined for eligibility using pre-determined criteria.

At this stage, we decided to drop the study arm for unstructured format due to very small quantity and volume of journals adopting this format, and therefore the difficulty to obtain a representative sample of adequate size for this group.

##### Data extraction

In addition to those items extracted in Part 1, we extracted the following information from each included abstract: type of journal, publication year, and geographical origin of the first author. Also, from the full-texts of included abstracts, we extracted number of centres (single centre/multi-centre) and the existence of financial support (funded/non-funded). One author (F.H.) carried out the data extraction and examined all extracted data with other authors.

##### Assessment of reporting quality

After sample creation, all included abstracts were collated into a Word document with journal title, author names and affiliations removed to allow for blinded quality assessment. F.H. and one of the other authors (T.W., A.M.G., H.W.) assessed the quality of methodology reporting of each included abstract independently and in duplicate, using the scoring criteria (Additional file 2) refined during pilot study. T.W., A.M.G. and H.W. assessed the same amount of HS and IMRaD abstracts, which were assigned randomly using an online random number generator. In addition to those 9 quality items, the scores of which contributed to our primary outcome (OQS), we also documented the reporting of 4 supplementary items (Table 1). All discrepancies were resolved by discussions.

### Statistical analyses

In Part 1 of this study, we used descriptive statistics to summarise the usage of structure formats and relevant editorial policies by journal and other characteristics. We also used the *Groups* command of Stata (version 14.1; StataCorp, College Station, TX, USA) to analyse the combination pattern of heading terms.

For Part 2, firstly, we performed both unadjusted (linear regression) and adjusted (generalised estimation equation, GEE; primary analysis) univariable and multivariable analyses to investigate the association between OQS (dependent variable) and structure format. Potential confounders, namely type of journal, continent of origin, publication year, number of centres, and existence of financial support were also analysed as independent variables, since previous literature suggested significant association between these factors and the reporting quality of RCT abstracts [24–27, 33]. As determined a priori, we entered all explanatory variables with *p* < 0.1 in univariable analyses into multivariable modelling. In linear regressions, no significant violation of normality was indicated in assessments of residuals. Tolerance and the variance inflation factor (VIF) were used to detect multicollinearity; any explanatory variable with a tolerance below 0.1 or VIF above 10 would be excluded from the final model [49]. In GEEs, we set *journal* as the grouping factor to account for potential clustering effects among abstracts published in the same journal; for this continuous outcome, we used a linear model with semi-robust standard errors and an exchangeable correlation matrix.

Secondly, we compared the IMRaD and HS groups in the reporting of each quality/supplementary item, using crude odds ratios (ORs) and adjusted ORs derived from GEEs. In GEEs, again we set *journal* as the grouping factor to take account of potential clustering effects among abstracts published in the same journal; for these binary outcomes, we adopted a binary logistic model with semi-robust standard errors and an exchangeable working correlation matrix. For all statistical analyses, a two-sided *p* < 0.05 was set as the criterion for statistical significance.

#### Ancillary analyses

In this study, we used a relatively broad definition for the HS format, which did not require the usage of all the 8 headings proposed by Haynes et al. [9]. To test the robustness of our results, we carried out a post hoc sensitivity analysis by further dividing the HS group into the following two groups, and repeating our analyses on the association between OQS and structure format (i.e. linear regressions and GEEs).

- 8-heading group: abstracts that incorporated all the eight headings proposed by Haynes et al. [9];
- Other HS group: abstracts that did not incorporate all eight headings but still fulfilled our definition for HS abstracts.

## Results

### Part 1: Structure format usage and journal policies

#### Characteristics of included journals and abstracts

**Fig. 1:**
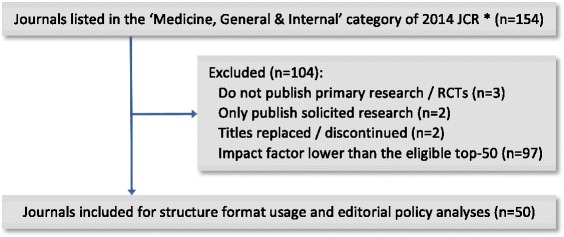
Flow diagram for journal selection. * Thomson Reuters 2014 Journal Citation Report ® Science Edition

As pre-planned, we selected fifty journals from the 154 listed in the ‘Medicine, General & Internal’ category of 2014 JCR (Table 2, Fig. 1) [42]. Among these, twenty-four were general medical journals, while the other 26 were specialty journals focused on internal medicine or more specific fields (e.g. family/preventive/pain medicine). According to the JCR, the impact factor of these journals ranged widely from 1.698 to 55.873 [42].

**Table 2:** Abstracts of RCTs published in top-50 journals in the ‘Medicine, General and Internal’ category during July–December 2015 - Frequency of structure formats and relevant editorial policies^a^ by journal (*n* = 370)

No.	Journal	N of RCTs identified (%)	Structure format used (%)			Structure format required	Specific instructions for each heading	Word limit	CONSORT for Abstracts endorsement
			Unstructured (*n* = 46)	IMRaD (*n* = 215)	HS (*n* = 109)				
1	New England Journal of Medicine	73 (19.7)	0 (0.0)	73 (100.0)	0 (0.0)	IMRaD	No	250	Not mentioned
2	The Lancet	53 (14.3)	0 (0.0)	53 (100.0)	0 (0.0)	IMRaD	Yes	300	Required
3	JAMA - Journal of American Medical Association	38 (10.3)	0 (0.0)	0 (0.0)	38 (100.0)	HS	Yes	350	Not mentioned
4	Annals of Internal Medicine	7 (1.9)	0 (0.0)	0 (0.0)	7 (100.0)	HS	No	275	Not mentioned
5	BMJ - British Medical Journal	8 (2.2)	0 (0.0)	4 (50.0)	4 (50.0)	HS	Yes	400	Required
6	PLOS Medicine	4 (1.1)	0 (0.0)	4 (100.0)	0 (0.0)	IMRaD	Yes	No limit	Not mentioned
7	JAMA Internal Medicine	7 (1.9)	0 (0.0)	0 (0.0)	7 (100.0)	HS	Yes	350	Not mentioned
8	BMC Medicine	6 (1.6)	0 (0.0)	6 (100.0)	0 (0.0)	IMRaD	Yes	350	Recommended
9	Journal of Cachexia Sarcopenia and Muscle	0 (0)	–	–	–	IMRaD	No	400	Not mentioned
10	Mayo Clinic Proceedings	2 (0.5)	0 (0.0)	2 (100.0)	0 (0.0)	IMRaD	No	250	Not mentioned
11	Journal of Internal Medicine	1 (0.3)	0 (0.0)	0 (0.0)	1 (100.0)	IMRaD	No	250	Not mentioned
12	Canadian Medical Association Journal	1 (0.3)	0 (0.0)	1 (100.0)	0 (0.0)	IMRaD	Yes	250	Not mentioned
13	Medicine (Baltimore)	42 (11.4)	42 (100.0)	0 (0.0)	0 (0.0)	Not specified	–	350	Not mentioned
14	Annals of Family Medicine	4 (1.1)	0 (0.0)	4 (100.0)	0 (0.0)	IMRaD	No	250	Not mentioned
15	Translational Research	2 (0.5)	2 (100.0)	0 (0.0)	0 (0.0)	Unstructured	–	250	Not mentioned
16	American Journal of Medicine	6 (1.6)	0 (0.0)	6 (100.0)	0 (0.0)	IMRaD	No	250	Not mentioned
17	American Journal of Preventive Medicine	11 (3.0)	0 (0.0)	4 (36.4)	7 (63.6)	HS	No	300	Not mentioned
18	Medical Journal of Australia	2 (0.5)	0 (0.0)	0 (0.0)	2 (100.0)	HS	Yes	250	Not mentioned
19	Annals of Medicine	0 (0)	–	–	–	IMRaD	No	200	Not mentioned
20	Deutsches Arzteblatt International	5 (1.4)	0 (0.0)	5 (100.0)	0 (0.0)	IMRaD	No	200	Not mentioned
21	Journal of General Internal Medicine	12 (3.2)	0 (0.0)	4 (33.3)	8 (66.7)	HS	No	300	Not mentioned
22	Preventive Medicine	8 (2.2)	0 (0.0)	7 (87.5)	1 (12.5)	Unstructured	–	250	Not mentioned
23	European Journal of Internal Medicine	1 (0.3)	0 (0.0)	1 (100.0)	0 (0.0)	Not specified	–	250	Not mentioned
24	Palliative Medicine	0 (0)	–	–	–	HS	Yes	Not specified	Not mentioned
25	Journal of Pain and Symptom Management	4 (1.1)	0 (0.0)	4 (100.0)	0 (0.0)	IMRaD	No	250	Not mentioned
26	American Journal of Chinese Medicine	0 (0)	–	–	–	Unstructured	–	250	Not mentioned
27	European Journal of Clinical Investigation	1 (0.3)	0 (0.0)	1 (100.0)	0 (0.0)	IMRaD	No	250	Not mentioned
28	Current Medical Research and Opinion	10 (2.7)	0 (0.0)	8 (80.0)	2 (20.0)	HS	Yes	300	Not mentioned
29	Internal and Emergency Medicine	1 (0.3)	1 (100.0)	0 (0.0)	0 (0.0)	Not specified	–	250	Not mentioned
30	International Journal of Clinical Practice	5 (1.4)	1 (20.0)	4 (80.0)	0 (0.0)	Not specified	–	Not specified	Not mentioned
31	QJM - An International Journal of Medicine	0 (0)	–	–	–	HS	No	250	Not mentioned
32	Pain Medicine	10 (2.7)	0 (0.0)	5 (50.0)	5 (50.0)	HS	No	250	Not mentioned
33	Journal of Hospital Medicine	0 (0)	–	–	–	HS	No	250	Not mentioned
34	British Journal of General Practice	3 (0.8)	0 (0.0)	0 (0.0)	3 (100.0)	HS	No	250	Not mentioned
35	BMJ Open	21 (5.7)	0 (0.0)	2 (9.5)	19 (90.5)	HS	Yes	300	Recommended
36	American Journal of Managed Care	5 (1.4)	0 (0.0)	0 (0.0)	5 (100.0)	HS	Yes	250	Not mentioned
37	Polish Archives of Internal Medicine	0 (0)	–	–	–	IMRaD	No	250	Not mentioned
38	Journal of the Royal Society of Medicine	0 (0)	–	–	–	HS	No	300	Not mentioned
39	Swiss Medical Weekly	0 (0)	–	–	–	IMRaD	No	600	Not mentioned
40	Journal of Women’s Health	2 (0.5)	0 (0.0)	2 (100.0)	0 (0.0)	IMRaD	No	250	Not mentioned
41	Archives of Medical Science	3 (0.8)	0 (0.0)	3 (100.0)	0 (0.0)	IMRaD	No	250	Not mentioned
42	AMYLOID - Journal of Protein Folding Disorders	0 (0)	–	–	–	Not specified	–	200	Not mentioned
43	International Journal of Medical Sciences	4 (1.1)	0 (0.0)	4 (100.0)	0 (0.0)	Not specified	–	Not specified	Not mentioned
44	Journal of the American Board of Family Medicine	3 (0.8)	0 (0.0)	3 (100.0)	0 (0.0)	IMRaD	No	Not specified	Not mentioned
45	Upsala Journal of Medical Sciences	0 (0)	–	–	–	IMRaD	No	250	Not mentioned
46	Netherlands Journal of Medicine	0 (0)	–	–	–	IMRaD	Yes	250	Not mentioned
47	Journal of the Formosan Medical Association	2 (0.5)	0 (0.0)	2 (100.0)	0 (0.0)	IMRaD	Yes	250	Not mentioned
48	Journal of Urban Health	0 (0)	–	–	–	Unstructured	–	Not specified	Not mentioned
49	Family Practice	0 (0)	–	–	–	IMRaD	No	Not specified	Not mentioned
50	Postgraduate Medicine	3 (0.8)	0 (0.0)	3 (100.0)	0 (0.0)	IMRaD	No	300	Not mentioned

IMRaD: introduction, methods, results, and discussion format; HS: highly structured format;

CONSORT for Abstracts: the CONSORT (Consolidated Standards of Reporting Trials) extension guidelines for reporting of RCT abstracts [23]

^a^Editorial policies according to journals’ ‘instructions to authors’ (as of April 2016)

Through hand-searches, we identified a total of 370 RCT abstracts from 36 of the included journals, published during July–December 2015. No RCT was found from the other 14 journals during the same period. Most of the identified abstracts were published in general medical journals (77.0%) (Additional file 3: Table S1), mainly the *New England Journal of Medicine* (19.7%), *Lancet* (14.3%), *Medicine (Baltimore)* (11.4%), *JAMA* (10.3%), and *BMJ Open* (5.7%) (Table 2).

#### Actual usage of structure formats

Of all identified RCT abstracts, 46 (12.4%) were in the unstructured format, 215 (58.1%) IMRaD and 109 (29.5%) HS. About half (50.5%) of the HS abstracts incorporated all the eight headings proposed by Haynes et al. [9] (Additional file 3: Table S1). Among 36 journals that published RCTs during July – December 2015, three journals only used the unstructured format, 18 only used IMRaD, seven only used HS, and the other eight used two different structure formats (Table 2). The proportions of structured (98.8% vs. 84.2%) and HS (51.8% vs. 22.8%) abstracts were both higher in specialty journals than in general journals. However, among HS abstracts that adopted all the Haynes 8 headings [9], the majority (87.3%) were from general medical journals (Additional file 3: Table S2).

The average overall word count of HS (343.8) and IMRaD (340.6) abstracts were comparable, but both higher than unstructured abstracts (268.5). HS abstracts used on average approximately 130 words in methods sections, while for IMRaD and unstructured formats this figure was about 110 and 80, respectively. In addition, according to the median values, a typical HS abstract was written in 8 sections using 10 heading terms, with 3 of the sections and 5 of the heading terms describing trial methodology. Whereas a typical IMRaD abstract used a total of 4 paragraphs and 4 heading terms, with only 1 paragraph and 1 heading term regarding methods (Additional file 3: Table S1).

A total of 39 different heading terms were identified from the included structured abstracts. The usage frequency of each heading term is listed in Additional file 3: Table S3. In addition, Additional file 3: Table S4 shows the most frequent patterns of heading term combinations. The most frequent pattern was “*Background*, *Method(s)*, *Results*, *Conclusion(s)*” in IMRaD abstracts, and “*Importance*, *Objective(s)*, *Design*, *Setting*, *Participants*, *Interventions*, *Main outcome measure(s)*, *Results*, *Conclusions*, *Relevance*, *Trial registration*” in HS abstracts.

#### Relevant editorial polices

Among those fifty included journals, four required the unstructured format, 24 required IMRaD, and 16 required HS for abstracts of original/primary research. The other six journals did not state any requirement on abstract structure format in their ‘instructions to authors’. In addition, half of journals requiring the HS format and one quarter of those requiring IMRaD gave instructions on the content to be reported under each heading that they required, although the amount of detail in such instructions varied greatly between journals (Table 2).

In terms of word limit, the most common requirement was 250 words, which was adopted by 25 journals. One journal (*PLOS Medicine*) set no limit to the length of abstracts, and another six journals did not specify any word limit for abstracts in their journal instructions. Additionally, only four journals mentioned the CONSORT for Abstracts guidelines [23] in their ‘instructions to authors’: *The Lancet* and *BMJ* required the use of these guidelines, while *BMC Medicine* and *BMJ Open* recommended them (Table 2).

### Part 2: Structure format and methodology reporting quality

#### Sample creation

**Fig. 2:**
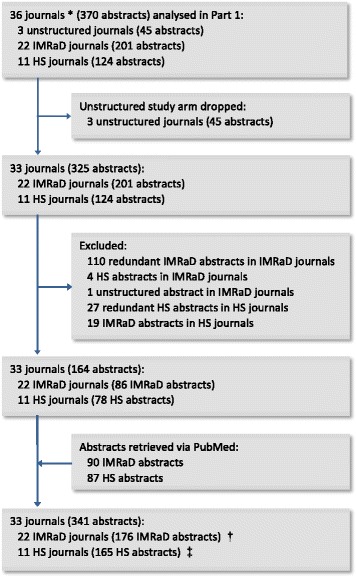
Flow diagram for study Part 2. * Journals with at least 1 RCT identified in study Part 1. † 8 abstracts included from each of the 22 IMRaD journals (journal no.1, 2, 6, 8, 10, 11, 12, 14, 16, 20, 22, 23, 25, 27, 28, 30, 40, 41, 43, 44, 47, 50 in Table 2). ‡ 15 abstracts included from each of the 11 HS journals (journal no.3, 4, 5, 7, 17, 18, 21, 32, 34, 35, 36 in Table 2)

During our PubMed searches for previous RCTs, we found it difficult to retrieve enough HS RCT abstracts for one HS journal (*Journal of Internal Medicine*), which published one RCT abstract in the HS format during July–December 2015 but required IMRaD in its journal instructions. We re-categorised this journal as an IMRaD journal, which resulted in a total of 22 IMRaD journals and 11 HS journals. Therefore, according to the calculated sample size (162 for each group), we included a total of 341 abstracts: 176 IMRaD abstracts (8 from each IMRaD journal) and 165 HS abstracts (15 from each HS journal) (Fig. 2).

#### Characteristics of included abstracts

As shown in Additional file 3: Table S5, most of the included abstracts were describing funded (87.4%), multi-centre (60.1%) trials, published in 2015 (76.0%), and by authors from Europe (37.0%) or North America (37.0%). About half of them were from general medical journals, while the other half from medical specialty journals. In addition, among abstracts in the HS format, only 69 (41.8%) incorporated all the eight headings proposed by Haynes et al. [9].

#### Structure format and overall reporting quality

The mean OQS (SD) was 4.41 (1.39) for the overall sample, 4.10 (1.48) for the IMRaD group and 4.75 (1.19) for the HS group. According to linear regression analyses, the OQS of the HS group was significantly higher than that of the IMRaD group both before (*B* = 0.66; 95% CI: 0.37 to 0.94; *p* < 0.001) and after (*B* = 0.69; 95% CI: 0.40 to 0.99; *p* < 0.001) potential confounders were taken into account (Additional file 3: Table S6). GEE analyses also showed that the OQS of HS abstracts were significantly higher than IMRaD abstracts in both univariable (*B* = 0.66; 95% CI: 0.15, 1.16; *p* = 0.011) and multivariable (*B* = 0.54; 95% CI: 0.06 to 1.03; *p* = 0.028) analyses (Table 3).

**Table 3:** Association between quality of methodology reporting, structure formats and potential confounders - Univariable and multivariable generalised estimation equation (GEE) derived coefficients (*B*) and 95% confidence intervals, with overall quality score (OQS) as the dependent variable and journal as the grouping factor (*n* = 341 abstracts from 33 journals)

		Univariable				Multivariable^b^		
Explanatory variables	Category/unit	*B*	95% CI	*P* value	QICC^a^	*B*	95% CI	*P* value
*Structure format*	IMRaD	Reference				Reference		
	HS	0.66	(0.15, 1.16)	0.011	620.2	0.54	(0.06, 1.03)	0.028
*Journal type*	General	Reference						
	Specialty	−0.30	(−0.86, 0.25)	0.285	650.3			
*Continent*				0.838	651.0			
	Europe	Reference						
	North America	−0.16	(−0.55, 0.22)	0.408				
	Asia	−0.28	(−0.86, 0.31)	0.353				
	Oceania	0.20	(−0.63, 1.03)	0.638				
	Others	−0.23	(−1.29, 0.84)	0.677				
*Publication year*	1 year	0.09	(−0.06, 0.25)	0.224	652.7			
*No. of centres*	Single centre	Reference				Reference		
	Multi-centre	0.42	(0.16, 0.68)	0.002	623.9	0.36	(0.08, 0.64)	0.013
*Funded*	No	Reference				Reference		
	Yes	0.69	(0.39, 0.99)	<0.001	625.5	0.57	(0.24, 0.90)	0.001

*IMRaD* Introduction, methods, results, and discussion format, *HS* Highly structured format

^a^QICC, Corrected quasi likelihood under independence model criterion

^b^For the final multivariable model, intercept = 3.437, QICC = 580.3

#### Structure format and reporting of each item

Most of the included abstracts adequately reported random allocation (98.5%), eligibility criteria for participants (95.9%), details of interventions (78.6%), and the unit of randomisation (72.1%). However, the reporting of study setting (53.4%) and primary/main outcome (51.9%) was only adequate in about half of the abstracts. Forty percent abstracts provided the time point when the primary/main outcomes were assessed. Only about one third (35.2%) and one fifth (21.1%) of all abstracts adequately reported trial design and blinding, respectively. Less than 5% reported the methods used for random sequence generation (4.7%) or allocation concealment (2.1%). In addition, the number of described primary/main outcomes ranged widely from 0 to 69, with a median of 1 and an interquartile range (IQR, 25th to 75th percentile) of 0 to 1 (Table 4).

**Table 4:** Reporting of each quality item and supplementary item by structure format, presented with unadjusted and adjusted odds ratios (ORs)

Items/Supplementary items	N (%)			Crude OR (95% CI)	Adjusted OR (95% CI); *P* value^a^
	Overall (*n* = 341)	IMRaD (*n* = 176)	HS (*n* = 165)		
1. Design	120 (35.2)	54 (30.7)	66 (40.0)	1.51 (0.96, 2.36)	1.51 (0.77, 2.93); 0.228
2. Participant	327 (95.9)	166 (94.3)	161 (97.6)	2.43 (0.75, 7.89)	2.43 (0.80, 7.33); 0.116
3. Setting	182 (53.4)	65 (36.9)	117 (70.9)	4.16 (2.64, 6.56)	4.16 (1.74, 9.97); 0.001
4. Interventions	268 (78.6)	138 (78.4)	130 (78.8)	1.02 (0.61, 1.72)	1.02 (0.64, 1.62); 0.924
5. Outcome	177 (51.9)	72 (40.9)	105 (63.6)	2.53 (1.63, 3.91)	2.53 (1.30, 4.92); 0.006
*5a. Time point*	138 (40.5)	49 (27.8)	89 (53.9)	3.04 (1.94, 4.76)	3.04 (1.49, 6.19); 0.002
*5b. No. of outcomes* ^b^	123 (36.1)^c^	93 (52.8)^c^	30 (18.2)^c^	1.87 (0.88, 3.97)^f^	1.83 (0.69, 4.87); 0.224
	177 (51.9)^d^	72 (40.9)^d^	105 (63.6)^d^		
	41 (12.0)^e^	11 (6.3)^e^	30 (18.2)^e^		
6. Random assignment	336 (98.5)	172 (97.7)	164 (99.4)	3.81 (0.42, 34.48)	3.81 (0.47, 30.76); 0.209
*6a. Unit of randomisation*	246 (72.1)	141 (80.1)	105 (63.6)	0.43 (0.27, 0.71)	0.43 (0.25, 0.77); 0.004
7. Sequence generation	16 (4.7)	11 (6.3)	5 (3.0)	0.47 (0.16, 1.38)	0.47 (0.10, 2.25); 0.343
8. Allocation concealment	7 (2.1)	5 (2.8)	2 (1.2)	0.42 (0.08, 2.19)	0.42 (0.06, 2.76); 0.366
9. Blinding (Masking)	72 (21.1)	38 (21.6)	34 (20.6)	0.94 (0.56, 1.59)	0.94 (0.42, 2.14); 0.887
*9a. Generic blinding*	57 (16.7)	28 (15.9)	29 (17.6)	1.13 (0.64, 1.99)	1.13 (0.51, 2.47); 0.765

*IMRaD* Introduction, methods, results, and discussion format, *HS* Highly structured format

^a^Derived from GEE analyses adjusting for potential clustering effect among abstracts published in the same journal, with individual quality item as the dependent variable (adequately reported vs. not adequately reported) and journal as the grouping factor;

^b^A continuous variable, not dichotomous;

^c^N (%) for category No. = 0; ^d^N (%) for category 1 ≤ No. ≤ 2; ^e^N (%) for category No. > 2;

^f^Derived from binary logistic regression (reference group: IMRaD format; dependent variable coding: [0] 1 ≤ No. ≤ 2, [1] No. > 2)

According to both crude ORs and adjusted ORs (aORs), the reporting of two quality items and two supplementary items was significantly different between the IMRaD and HS groups. Study setting (aOR = 4.16; 95% CI: 1.74 to 9.97; *p* = 0.001) and main outcome measure (aOR = 2.53; 95% CI: 1.30 to 4.92; *p* = 0.006) were reported significantly better in HS abstracts. More HS abstracts provided the time point for primary/main outcome assessment (aOR = 3.04; 95% CI: 1.49 to 6.19; *p* = 0.002), whereas more IMRaD abstracts described the unit of randomisation (aOR = 0.43; 95% CI: 0.25 to 0.77; *p* = 0.004) (Table 4).

#### Sensitivity analysis

After further dividing HS abstracts into two groups (8-heading/Other HS), multivariable linear regression suggested that both the 8-heading group (*B* = 0.77; 95% CI: 0.41 to 1.14; *p* < 0.001) and Other HS group (*B* = 0.62; 95% CI: 0.26 to 0.98; *p* = 0.001) had significantly higher OQS than the IMRaD group (Additional file 3: Table S7). However, according to multivariable GEE analysis, only the OQS of the 8-heading group (*B* = 0.75; 95% CI: 0.24 to 1.26; *p* = 0.004) was significantly higher than the IMRaD group (Additional file 3: Table S8).

## Discussion

### Principal findings

Part 1 of this study, based on a cross-sectional analysis of the instructions of fifty leading general medical/internal medicine journals and all RCT abstracts published in these journals during a 6-month period, provides insights into the current usage of structure formats and adoption of relevant editorial policies. Our results show that approximately 90% of all abstracts were structured, among which the IMRaD format was twice as common as the HS format. According to journals’ ‘instructions to authors’, about 80% of all selected journals required structured abstracts, with about 50% requiring IMRaD and the other 30% requiring HS. However, only 14 journals (28.0%) specified what should be reported under each heading that they required, and only four journals (8.0%) required or recommended the use of CONSORT for Abstracts guidelines.

In Part 2, we compared 341 IMRaD and HS abstracts retrieved from 33 leading general medical/internal medicine journals, and found evidence that trial methodology is significantly better reported among RCT abstracts in the HS format than those in the IMRaD format. After taking into account potential confounders and clustering effects, the average OQS of HS abstracts was 0.54 higher than IMRaD abstracts (*p* = 0.028). Such an advantage could mainly be attributed to the better reporting of two quality items in HS abstracts: *setting* (*p* = 0.001) and *outcome* (*p* = 0.006). HS abstracts also reported the supplementary item *time point* better (*p* = 0.002). But more IMRaD abstracts provided the unit of randomisation (*p* = 0.004). In addition, for both HS and IMRaD abstracts the reporting of most quality and supplementary items needs improvement, especially for *allocation concealment*, *sequence generation*, *blinding* and *design*.

## Conclusions

In summary, for RCT abstracts, the IMRaD format is more frequently used and required by leading generalmedical/internal medicine journals than the HS format. Abstracts in the HS format report trial methodologymore completely than those in the IMRaD format.

### Comparison with other studies

#### Structure format usage and journal policies

Nakayama and colleagues [19] analysed the structure format of 304 original research abstracts published in the top-30 general medical/internal medicine journals in 2001. They found that 61.8% abstracts were structured, among which 66.5% used the IMRaD format and 33.5% used 8-heading (or its variations). Also, they examined the top-30 journals’ requirements on abstracts using their ‘instructions to authors’. The instructions of 27 journals were available, of which 13 (48.1%) required the IMRaD format, eight (29.6%) required 8-heading (or its variations), while the other 6 (22.2%) had no specific requirement. In comparison, results of our study suggest a much higher proportion of structured abstracts (87.6%), but a very similar 2:1 ratio between the number of IMRaD and HS abstracts, and similar proportions of journals requiring each structure format.

However, readers should note the methodological differences between Nakayama et al. s [19] study and ours. First, Nakayama et al. [19] did not provide their definitions for IMRaD and 8-heading formats, or the methods used to categorise borderline abstracts such as those with between 4 and 8 main headings. To overcome such ambiguity and ensure replicability, we decided to use the broader concept HS for our main study design and to analyse/report information related to the Haynes eight headings [9] separately. Second, the abstracts and journals analysed in these two studies were different. Aside from differences in publication date (2001 vs. 2015) and number of selected journals (top-30 vs. top-50), Nakayama et al. [9] looked at abstracts of all original research while our study was focused on RCT abstracts only. To our knowledge, no previous study has analysed specifically the structure format of RCT abstracts published in leading medical journals. In addition, since the 8-heading format was originally intended for the reporting of clinical trials [10–12], difficulty in adopting the 8-heading (and other HS) format could be different between trial abstracts and abstracts describing other types of original studies.

Endorsement of the CONSORT for Abstracts guidelines in journals’ ‘instructions to authors’ is recommended by the CONSORT group [23]. A study by Hopewell et al. [50] provided evidence that endorsement of these guidelines, when combined with active editorial policies to implement them, can lead to improvements in the reporting of RCT abstracts. Previously, a number of studies have investigated the endorsement level of reporting guidelines in the ‘instructions to authors’ of medical journals [44–46, 51–55]. However, to our knowledge, only two studies have looked at the endorsement of CONSORT for Abstracts guidelines in author instructions, reporting that these guidelines were mentioned by only five (4.6%) out of 109 main dental journals (as of May 2015) [46] and 11 (6.5%) out of 168 high-impact medical journals (as of December 2014) [55]. One recent study, aimed at assessing the reporting of RCT abstracts in top-5 general medical journals, reported that only *The Lancet* and *BMJ* mentioned these guidelines in their ‘instructions to authors’ [36]. Our study shows similar results in this respect and indicates that, since *The Lancet* and *BMJ* added the CONSORT for Abstracts guidelines into their author instructions in January 2008 [50] very few other medical journals have adopted the same policy.

#### Structure format and methodology reporting quality

The present study is, to our knowledge, the first study designed to compare the reporting quality of IMRaD and HS RCT abstracts. Among previous studies that used the CONSORT for Abstracts checklist to assess RCT abstract reporting, five have taken into account the structure of abstracts as a confounding variable [24–28]. In four of these studies, structure was analysed in two categories (unstructured vs. structured): 3 studies found that the reporting quality was significantly higher in structured abstracts, but not significantly higher anymore when other explanatory variables were accounted for [24–26]; while in the other study, authors found no significant difference between structured and unstructured abstracts in univariable analysis [27]. In a more recent study, Bigna et al. [28] categorised abstract structure into IMRaD, 8-heading and ‘one-block’. They found that both IMRaD and 8-heading abstracts were significantly better reported than ‘one-block’ abstracts in univariable GEE analysis, and again the difference was not significant anymore when other covariates were adjusted for. However, Bigna et al. [28] also did not make any direct comparison between IMRaD and 8-heading abstracts.

Interpretation of the differences between our results and those of the above-mentioned studies is complicated by major differences in study design and methodology. First, the dependent variable used in all above-mentioned studies was the overall quality of abstract reporting (including the reporting of methodology, results, interpretation, registration and other aspects), while our study was focused on the reporting of trial methodology only. Second, the covariates accounted for in multivariable analyses varied greatly among these studies, making the results of these analyses not fully comparable at least. Third, in previous studies structure format was treated as a potential confounder instead of the primary objective, therefore they might be underpowered to detect a meaningful difference between structured and unstructured abstracts. Additionally, differences in results could also be attributed to different sources of abstracts. Our study included abstracts from recent issues of leading general medical/internal medicine journals, while those previous studies assessed abstracts from other medical specialty journals [26, 27] or certain specific areas [24, 25, 28].

In this study, after analysing the quality of reporting for each item, we found that eligibility criteria for participants and details about intervention were adequately reported by most abstracts, whereas the reporting of *design*, *blinding*, *sequence generation* and *allocation concealment* was poor or even rare. This pattern is generally in keeping with the findings of four previous studies, which assessed the reporting quality of RCT abstracts from major general medical journals [31, 33, 36, 50].

In addition, since to our knowledge no former similar study provided explicit information on the reporting of unit of randomisation, time point of primary/main outcome assessment, and number of described primary/main outcomes, we assessed these aspects as supplementary items.

First, the unit of randomisation should be clearly reported so that readers can understand the trial design and assess its appropriateness [56–58]. Although reporting of the unit of randomisation was not recommended in the CONSORT for Abstracts guidelines [23], the CONSORT 2010 general guidelines did state that it is desirable to include such information in the abstract [59].

Secondly, the CONSORT for Abstracts asked in its Explanation article [23] that authors report the time point of primary outcome assessment, but such requirement was not mentioned in its checklist [60]. Most previous similar studies did not specify whether this information was required in their scoring criteria, thus in order to ensure clarity we have treated the definition of primary/main outcomes and the time point of assessment as two separate items.

Thirdly, the primary outcome is usually one predetermined outcome that is considered of greatest importance and used in the sample size calculation [59]. Although the use of multiple primary outcomes in a trial incurs interpretation problems and is not recommended by the CONSORT statement [59], according to an empirical study many recently published trials still used multiple primary outcomes. Moreover, in their abstracts, most of these trials either did not specify any outcome or specified multiple outcomes without distinguishing primary and secondary outcomes [61]. As both the CONSORT for Abstracts guidelines [23] and the Haynes proposal for structured abstracts [9] did not specify the appropriate number of primary outcomes to report in abstracts, in this study we have adopted an arbitrary standard (for the item *outcome*) that authors should define between 1 and 2 primary outcomes (e.g. one efficacy outcome and one safety outcome, or two efficacy outcomes of equal importance) in their abstracts, or 1 to 2 ‘main’ outcomes if the primary and secondary outcomes were not distinguished.

### Strengths and limitations

To our knowledge, this study is the first of its kind to: 1) compare the reporting quality of structured RCT abstracts in different formats (IMRaD vs. HS); 2) explore the usage of different structure formats in RCT abstracts published in leading general medical/internal medicine journals; 3) analyse the usage frequency and combination pattern of heading terms by structure format; and 4) determine the endorsement level of the CONSORT for Abstracts guidelines in the ‘instructions to authors’ of leading general medical/internal medicine journals. Furthermore, based on the CONSORT for Abstracts guidelines, we developed and used a checklist including 9 quality items and 4 supplementary items specifically for the assessment of RCT abstract methodology reporting. A large number of leading journals were selected and analysed. An internal pilot study was conducted to help ensure the quality of main study. Besides, potential clustering effects and confounding factors supported by the previous literature were taken into account in our study design and statistical analyses.

Our study has some limitations. First, although we included about one-thirds of all journals listed under the ‘Medicine, General & Internal’ category of 2014 JCR, a number that is much higher than most previous similar studies, our findings may not be representative of other general medical/internal medicine journals or journals in other medical specialties. It is feasible that the reporting quality of RCT abstracts in lower impact journals and medical specialty journals would be lower than our results [28, 62]. But whether our findings regarding structure format and reporting quality also apply to other journals/specialties remains to be studied.

Secondly, in part 2 of our study, we could not obtain a representative and adequately sized group of unstructured RCT abstracts due to the fact that the unstructured format is not widely used now in leading general medical/internal medicine journals. However, both the CONSORT group [23] and the International Committee of Medical Journal Editors (ICMJE) [1] have strongly recommended the use of structured abstracts. Plus, a very recent study, aimed at assessing the abstract reporting of HIV/AIDS RCTs, has shed some light on the difference between unstructured abstracts and abstracts in the IMRaD and 8-heading format, respectively [28].

Lastly, one limitation that our study shares with other similar studies [19, 28, 36] is that the journal instructions were collected at a date later than the publication of included RCT abstracts, which means that the documented journal requirements might be different from the version used during the editorial process of those included RCTs. As a result, we could not draw any tenable conclusion from a comparison between journals’ requirements and their actual usage of structure format, or use the endorsement level of CONSORT for Abstracts as a reasonable explanatory variable in statistical analyses. Future prospective studies are needed to provide insights into these aspects.

### Implications and recommendations

Thirty years after Haynes and colleagues proposed the use of structured abstracts and the 8-heading format [8, 9], most RCT abstracts in general medical/internal medicine journals are now structured but in a simpler IMRaD format. The results of our main study and sensitivity analysis indicate that HS abstracts, especially those that incorporated all eight headings proposed by Haynes et al. [9], have better reporting of trial methodology than IMRaD abstracts. The main reason for such advantage could be that those extra methodology headings included in the HS format can remind authors, peer reviewers and editors the necessity of providing relevant details in the abstract [9, 12].

However, in this study we also found that the reporting of *allocation concealment*, *sequence generation*, *blinding*, *design*, *outcome* and *setting* need improvement in both HS and IMRaD abstracts. Based on the findings of our study, as well as the Haynes proposal for structured abstracts [9] and the CONSORT for Abstracts guidelines [23], we propose a new 12-heading HS format specifically for the reporting of RCT abstracts (Table 5). We welcome comments from all relevant experts and stakeholders on this format, and look forward to feedback on its applicability and effectiveness after initial implementation.

**Table 5:** A 12-heading HS format recommended for the reporting of RCT abstracts, modified from the original Haynes proposal for structured abstracts [9] and the CONSORT for Abstracts guidelines [23]

Heading	Content instruction
1. Objective	State the specific objective or question addressed in the trial. If more than one objective is addressed, indicate the primary objective (based on the predetermined primary outcome) and key secondary objectives.
2. Design^a^	Use the term ‘randomised’ to indicate that this is an RCT; describe explicitly the design of the trial (e.g. parallel group, cluster randomised, crossover, factorial, superiority, equivalence or noninferiority, or a combination of these designs); report the duration of follow-up.
3. Setting^a^	Provide information about the trial setting, including the level of care (e.g. primary, secondary, tertiary care) and number of participating centres; describe the geographical location if important (e.g. population research in communities).
4. Participants and interventions^b^	Provide eligibility criteria for participants (e.g. demographics, clinical diagnosis, comorbid conditions) and details about the interventions for each group (e.g. dose, route and duration of administration, surgical procedure/technique, name of drug, manufacturer of inserted device, main content of education/lifestyle intervention activity); state the number of participants randomised to each group and the unit of randomisation;
5. Main outcome measure(s)^c^	Clearly state what the primary outcome was (i.e. the predetermined outcome considered of greatest importance and usually the one used in sample size calculation) and when it was assessed; describe key secondary outcome if important, make sure that primary outcome and secondary outcomes are distinguished; if the trial abstract focuses on a secondary outcome, identify both this outcome and the primary outcome.
6. Sequence generation^a^	Describe the methods used for random sequence generation (e.g. random number table, computer random number generator, coin tossing, minimisation).
7. Allocation concealment^a^	Describe the methods used for allocation concealment (e.g. central allocation, sequentially numbered identical containers, sequentially numbered opaque, sealed envelopes); state ‘None’ when no measures were taken to conceal allocation.
8. Blinding (masking)	State the blinded parties among participants, caregivers/personnel, data assessors and data analysts (automatically indicating that those unmentioned parties were not blinded); avoid generic descriptions (e.g. single-blind, double-blind); state ‘None’ if blinding was not used or not possible/appropriate in the trial.
9. Results	Describe the number of participants in each group that were included in the analysis; for the primary outcome, state a result for each group, the estimated effect size and its precision; report any important adverse events (if no adverse events occurred state this explicitly).
10. Conclusions	Give a general interpretation that is consistent with the trial results, with benefits and harms balanced.
11. Trial registration^a^	Provide the registration number and name of trial register.
12. Funding^a^	Report the source of funding.

*HS* Highly structured, CONSORT for Abstracts: the CONSORT (Consolidated Standards of Reporting Trials) extension guidelines for reporting of RCT abstracts [23]

^a^For brevity, content under these headings can be written in phrases rather than complete sentences

^b^Two Haynes headings ^9^ are combined together to facilitate the reporting of unit of randomisation and number of randomised participants, for example:

(1) Sixty patients with breast cancer of stage 0 to III were randomly assigned in a 2:1 ratio to receive surgery technique A (A group) or surgery technique B (B group);

(2) Four classes of healthy high school students were randomly allocated to two additional 40-min courses of outdoor activities (intervention group, 2 classes, 60 students) or their usual pattern of activity (control group, 2 classes, 52 students)

^c^Using multiple primary outcomes in a trial incurs interpretation problems and is not recommended [59]

To most stakeholders of medical research, including clinicians [5, 6], editors [7] and policy-makers [63], the abstract of a medical research report serves as an important and potentially the sole basis for their judgments of the study’s reliability and applicability. In addition, abstracts are often the only part of medical research articles that readers have access to, especially those in low-income countries and resource-poor institutions [2, 3]. However, the reporting quality of RCT abstracts published in medical journals has been suboptimal [24, 26–28, 31]. A recent time series analysis showed that, according to the current trend of improvement, it would take about 50 years for RCT abstracts in major paediatric journals to report all items required in the CONSORT for Abstracts checklist [35].

In light of these, more efforts are needed from all stakeholders to further improve the reporting of RCT abstracts, and thereby reduce relevant avoidable research waste [64]. We recommend that the CONSORT group and the ICMJE consider endorsing the use of HS format, not just structured format, for RCT abstracts. We also recommend that journal editors require in their journal instructions that authors of RCTs use an appropriate HS format for their abstracts and adhere to the CONSORT for Abstracts guidelines [23].

## Additional files

Additional file 1: Study Protocol. (DOCX 52 kb)

Additional file 2: Scoring Criteria for the Assessment of Methodology Reporting. (DOCX 26 kb)

Additional file 3: Table S1. Abstracts of RCTs published in top-50 journals in the ‘Medicine, General and Internal’ category during July–December 2015 - Characteristics by structure format. **Table S2.** Abstracts of RCTs published in top-50 journals in the ‘Medicine, General and Internal’ category during July–December 2015 - Characteristics by type of journal. **Table S3.** Usage frequency of each heading term among identified RCTs with structured abstracts (*n* = 324). **Table S4.** Most frequent (>2%) patterns of heading term combinations among identified RCTs with structured abstracts (*n* = 324). **Table S5.** Characteristics of abstracts included for reporting quality assessment. **Table S6.** Association between quality of methodology reporting, structure formats and potential confounders - Univariable and multivariable linear regression derived coefficients (*B*) and 95% CIs, with overall quality score (OQS) as the dependent variable (*n* = 341). **Table S7.** Association between quality of methodology reporting, structure formats and potential confounders - Sensitivity analysis testing the definition for HS format - Univariable and multivariable linear regression derived coefficients (*B*) and 95% CIs, with overall quality score (OQS) as the dependent variable (*n* = 341). **Table S8.** Association between quality of methodology reporting, structure formats and potential confounders - Sensitivity analysis testing the definition used for HS format - Univariable and multivariable generalised estimation equation (GEE) derived coefficients (*B*) and 95% confidence intervals, with overall quality score (OQS) as the dependent variable and journal as the grouping factor (*n* = 341 from 33 journals). (DOCX 54 kb)
