# Supplementary material for: Structure formats of randomised controlled trial abstracts: a cross-sectional analysis of their current usage and association with methodology reporting
Source: BMC Med Res Methodol. 2018 Jan 10;18:6. doi: 10.1186/s12874-017-0469-3 (PMC5761197; doi:10.1186/s12874-017-0469-3)
Supplement: Supplementary file 1 — Study Protocol. (DOCX 52 kb) [file 12874_2017_469_MOESM1_ESM.docx]

**Additional file 1**

**Study Protocol**

[April 2016]

Fang Hua, Tanya Walsh, Anne-Marie Glenny, Helen Worthington

1. **Objectives**

- Present the current requirement and actual usage of different abstract formats by the top-50 general medical/internal medicine journals;^1^
- Investigate the association between different structure formats of RCT abstracts and the reporting of methodological aspects;
- Based on the study results, discuss the necessity of further promotion and possible improvement of the original 8-heading highly structured format.^2^

1. **Definitions of Structure Formats**

- Unstructured format:

Abstracts presented in one paragraph, with no distinct, labeled sections (e.g. Introduction, Methods);^3^

- IMRaD format:

Structured abstracts with four distinct sections labeled with ***I****ntroduction/Background/Objective(s)*, *(Materials/Patients and)* ***M****ethods*, ***R****esults*, **a**nd ***D****iscussion/Conclusion(s)*, respectively, with or without other separate sections for trial registration and/or source of funding;

- Highly structured (HS) format:

Structured abstracts with more than four distinct, labeled sections and at least one of the 5 headings (*Design*, *Setting*, *Patients/Participants*, *Interventions*, *Main Outcome Measure*) proposed by Haynes et al ^2^ for methodology reporting, with or without other separate sections for trial registration and/or source of funding.

1. **Selection of Journals**
   1. Inclusion criteria:

Top-50 journals listed under the “Medicine, General & Internal” category of the 2014 Journal Citation Report,^4^ which publish RCTs.

- 1. Exclusion criteria:
- Inactive/discontinued journals;
- Journals that do not publish RCTs;
- Journals that only publish solicited research.
  1. Data extraction:

Full journal title, impact factor, rank in the category, URL of the journal’s official website.

1. **Survey Regarding Journal Requirements**
   1. Materials/source of information:

The “Instructions to Authors” of all included journals, retrieved from their official websites (in the form of PDF documents).

- 1. Data extraction (for each journal):

Journal type (general vs. specialty), structure format requirement (highly structured, IMRaD, unstructured, or no requirement), headings required, whether detailed instruction is given for each required heading, word limit for abstracts, endorsement level of the general CONSORT guidelines and the CONSORT for Abstracts guidelines (not mentioned, recommended, or required).^5^

1. **Survey Regarding the Actual Usage of Each Structure Format**
   1. Materials/source of information:

RCT reports published in the included journals from July to December 2015, identified through a hand-search of each journal’s official online archive.^6^ The hand-search will be carried out by two authors.

- 1. Data extraction (for each identified RCT):

Title of article, title of journal, publication date, structure format used, headings used.

1. **Internal Pilot Study**
   1. Purposes:

- Calibration of researchers in reporting quality assessment;
- Inform sample size calculation for the main study.
  1. Materials:

From the identified RCT abstracts (see section 5.1), use stratified random sampling (with each journal as a stratum) to randomly choose:^7^

- Twelve HS abstracts from journals that only or mainly used HS format;
- Twelve IMRaD abstracts from journals that only or mainly used IMRaD format;
- Twelve unstructured abstracts from journals that only or mainly used unstructured format.
  1. Quality assessment:

Four authors (F.H., T.W., A-M.G. & H.W.) will assess the 36 abstracts independently and in duplicate, using a 9-item checklist developed based on the CONSORT for Abstracts guidelines and relevant explanations.^8^ (**Table**)

For each quality item, a score of ‘1’ will be given when the item is adequately reported, and a score of ‘0’ when the reporting is inadequate. The primary outcome is the Overall Quality Score (OQS; range, 0 to 9), which is calculated for each abstract by totaling the scores for all 9 items.

After all four authors have completed their assessments, a face-to-face meeting will be held for the source and resolution of all inter-examiner discrepancies and application of the checklist. A unanimous set of assessment results for the 36 abstracts will be reached as the basis of sample size calculation.

**Table.** Quality checklist used in this study for the assessment of methodology reporting in RCT abstracts ^8^

| No. | Item | Description | Main Importance |
| --- | --- | --- | --- |
| 1 | **Design** | Description of the trial design | RCT identification |
| 2 | **Participant** | Eligibility criteria for participants | Generalizability |
| 3 | **Setting** | Settings where the data were collected | Generalizability |
| 4 | **Interventions** | Interventions intended for each group | Generalizability |
| 5 | **Outcome Definition** | Clearly defined primary outcome for this report | Selective reporting bias |
| 6 | **Random Assignment** | Clear statement that participants were allocated to groups in a random manner | RCT identification |
| 7 | **Sequence Generation** | Description of the method used for random sequence generation | Selection bias |
| 8 | **Allocation Concealment** | Description of the method used for allocation concealment | Selection bias |
| 9 | **Blinding** | Whether participants, care givers, and those assessing the outcome were blinded to group assignment | Detection bias and performance bias |

- 1. Sample size calculation for the main study:

Use the mean OQS and standard deviations of each group, and an assumed smallest effect of interest (e.g. mean difference of 0.5 in OQS) to calculate a minimum sample size for the main study. Then according to the non-central t-distribution (NCT) approach, inflate the calculated sample size by 8% (for pilot study sized 12 per arm, type I error 5%, power 80%) to account for the fact that a sample estimate of the variance, rather than the population variance, was used in the sample size calculation.^9^

1. **Main Study**
   1. Materials:

Obtain an appropriate study sample based on the sample size calculation, journals’ requirement and actual usage of each structure format, and the number of RCTs published in each journal from July to December 2015. Initially, divide the included journals into three groups:

- Journals only (or mainly) used, or required the use of HS format;
- Journals only (or mainly) used, or required the use of IMRaD format;
- Journals only (or mainly) used, or required the use of unstructured format.

Then for each group, designate a maximum number (***N***) of RCT articles to include from each journal so that the minimum sample size required can be obtained. For journals that published less than ***N*** RCTs during July - December 2015, we will retrieve recent RCTs published in those journals before July 2015 via PubMed (search term, the *journal title*[Journal] AND “randomized controlled trial[publication type]”). For journals that published more than ***N*** RCTs during 2015, randomly choose ***N*** RCTs from each journal.

- 1. Data extraction (for each included RCT):

Title of article, title of journal, publication date, structure format used, word count of the Methods section (or the equivalent part), geographical origin of first author, number of centers (single vs. multi-center), and funding as reported (funded vs. non-funded).^10-12^

- 1. Quality assessment

F.H. and one other author (T.W., A-M.G. or H.W.) will assess the reporting quality of each included abstract independently and in duplicate, using the checklist and application methods agreed in the pilot study. All discrepancies will be resolved by discussion.

- 1. Statistical analyses

To account for the potential clustering effects among abstracts published in the same journal, we will use generalized estimating equation (GEE) analyses to investigate the association between OQS and potential predictors (including the structure format). First, univariable GEE analyses will be used to identify factors (see sections 4.2 & 7.2) that potentially influence the OQS. Then, significant factors (P<0.1) in univariable analyses will be entered into a multivariable GEE analysis, together with the structure format, to determine the adjusted impact of structure format on OQS. In addition, chi-square tests will be used to compare the adequate reporting rate of each quality item by structure formats. Any 2-tailed P value less than 0.05 will be considered statistically significant.

**References**

1. Nakayama T, Hirai N, Yamazaki S, et al. Adoption of structured abstracts by general medical journals and format for a structured abstract. J Med Libr Assoc 2005;**93**(2):237-42.

2. Haynes RB, Mulrow CD, Huth EJ, et al. More informative abstracts revisited. Ann Intern Med 1990;**113**(1):69-76.

3. U.S. National Library of Medicine. Structured Abstracts. 2014. (accessed 15 April 2016) <https://www.nlm.nih.gov/bsd/policy/structured_abstracts.html>.

4. Thomson Reuters. 2014 Journal Citation Reports® Science Edition. 2015. (accessed 20 March 2016) <https://jcr.incites.thomsonreuters.com/JCRJournalHomeAction.action>.

5. Meerpohl JJ, Wolff RF, Niemeyer CM, et al. Editorial policies of pediatric journals: survey of instructions for authors. Arch Pediatr Adolesc Med 2010;**164**(3):268-72.

6. Hua F, Walsh T, Glenny AM, et al. Reporting quality of randomized controlled trial abstracts presented at European Orthodontic Society congresses. Eur J Orthod 2015:doi: 10.1093/ejo/cjv094.

7. Julious SA. Sample size of 12 per group rule of thumb for a pilot study. Pharm Stat 2005;**4**(4):287-91.

8. Hopewell S, Clarke M, Moher D, et al. CONSORT for reporting randomized controlled trials in journal and conference abstracts: explanation and elaboration. PLoS Med 2008;**5**(1):e20.

9. Whitehead AL, Julious SA, Cooper CL, et al. Estimating the sample size for a pilot randomised trial to minimise the overall trial sample size for the external pilot and main trial for a continuous outcome variable. Stat Methods Med Res 2015;**25**(3):1057-73.

10. Ghimire S, Kyung E, Lee H, et al. Oncology trial abstracts showed suboptimal improvement in reporting: a comparative before-and-after evaluation using CONSORT for Abstract guidelines. J Clin Epidemiol 2014;**67**(6):658-66.

11. Guo JW, Iribarren SJ. Reporting quality for abstracts of randomized controlled trials in cancer nursing research. Cancer Nurs 2014;**37**(6):436-44.

12. Hua F, Deng L, Kau CH, et al. Reporting quality of randomized controlled trial abstracts: survey of leading general dental journals. J Am Dent Assoc 2015;**146**(9):669-78.e1.

**Differences Between Protocol and Study Report**

[November 2016]

1. Due to limited resources, the searches for RCTs were conducted by only one author (FH). But the same author repeated the searching and screening process and verified the eligibility of all included RCTs.
2. During the data extraction stage of Study Part 1, it was decided *post hoc* to extract the following information from each identified abstract: overall word count, word count for Methods section (or its equivalent part), overall number of paragraphs, number of methodology paragraphs, overall number of heading terms, number of heading terms regarding methodology, and whether all the Haynes eight headings were used.
3. During the pilot study, we decided to document the reporting of three supplementary items (*time point*, *number of outcomes*, *unit of randomisation*), which did not affect the calculation of our pre-determined primary outcome (overall quality score).
4. During the data extraction stage of Study Part 2, it was decided *post hoc* to extract the following information from each included abstract: overall number of paragraphs, number of methodology paragraphs, overall number of heading terms, number of heading terms regarding methodology, and whether all the Haynes eight headings were used.
