# Supplementary material for: Structure formats of randomised controlled trial abstracts: a cross-sectional analysis of their current usage and association with methodology reporting
Source: BMC Med Res Methodol. 2018 Jan 10;18:6. doi: 10.1186/s12874-017-0469-3 (PMC5761197; doi:10.1186/s12874-017-0469-3)
Supplement: Supplementary file 2 — Scoring Criteria for the Assessment of Methodology Reporting. (DOCX 26 kb) [file 12874_2017_469_MOESM2_ESM.docx]

**Additional file 2**

**Scoring Criteria for the Assessment of Methodology Reporting**

- 1. **Design:**

Explicit description of the trial design, e.g. parallel group, cluster randomised, crossover, factorial, superiority, equivalence or noninferiority, split-mouth/face/scar, or some other combination of these designs.

- 1. **Participants:**

Eligibility criteria for participants. General description (e.g. “34 healthy subjects”, “patients with lung cancer”) and description in other parts of the abstract (e.g. title, background, objectives) are acceptable.

- 1. **Setting:**

Settings where the data were collected. For behavioural and population research, descriptions with geographical information only (e.g. an Asian community in Boston) is acceptable. For research on medical treatment, the level of care (primary, secondary, tertiary) should be explicitly stated or indicated, so that readers can determine the generalizability of the findings to their own setting.

- 1. **Interventions:**

Interventions intended for each group (any details about the interventions, e.g., the exact dose, route of administration, duration of administration, surgical procedure/technique, manufacturer of inserted device, or the main content of an education/lifestyle intervention activity). In a case of a placebo/blank control group, when details have been given for the other group(s), a simple description of “placebo/blank control group” is acceptable.

- 1. **Outcome:**

Clearly defined primary/main outcome(s) for this trial. The term “primary/main outcome/measure/end point” has to be used. The number of described primary/main outcomes should not exceed two. If the abstract focuses on a secondary outcome of a trial, the abstract should identify both this outcome and the primary outcome of the trial.

5.1 **Time point:**

When was the primary/main outcome assessed (e.g., the time frame over which it was measured). When there are no or too many (>2) mentioned primary/main outcomes, this supplementary item is scored as “0”.

5.2 **No. of outcomes:**

The number of described primary/main outcome measure(s). (Quantitative, not “1” vs. “0”)

- 1. **Random assignment:**
- Clear statement (in title and/or abstract) that participants were allocated to groups in a randomised manner. When only the term “randomised (controlled) trial” or “RCT” was used without further details, we assume that this “random” means random allocation and give a score of “1”.

6.1  **Unit of randomisation:**

Description of the unit of random allocation (e.g. patients, schools, communities).

- 1. **Sequence generation:**

Description of the method used for random sequence generation (e.g., use of computer or random number table).

- 1. **Allocation concealment:**

Description of the method used for allocation concealment (e.g. centralized or pharmacy-controlled randomisation, sequentially numbered opaque sealed envelopes).

- 1. **Blinding:**

Whether or not participants, caregivers, and those assessing the outcomes were blinded to group assignment. When the blinding situation for at least one of the above-mentioned parties is given, we assume that this indicates all other parties were not blinded and give a score of “1”. Generic description, such as “single-blind” and “double-blind”, is not acceptable. In the case that blinding is not possible/appropriate during the trial, authors should use terms such as “open-label” or “non-blinded”.

9.1 **Generic blinding:**

Only generic description was used for blinding (e.g. “single-blind”, “double-blind”).
