# Supplementary material for: Structure formats of randomised controlled trial abstracts: a cross-sectional analysis of their current usage and association with methodology reporting
Source: BMC Med Res Methodol. 2018 Jan 10;18:6. doi: 10.1186/s12874-017-0469-3 (PMC5761197; doi:10.1186/s12874-017-0469-3)
Supplement: Supplementary file 3 — Abstracts of RCTs published in top-50 journals in the ‘Medicine, General and Internal’ category during July–December 2015 - Characteristics by structure format. Table S2. Abstracts of RCTs published in top-50 journals in the ‘Medicine, General and Internal’ category during July–December 2015 - Characteristics by type of journal. Table S3. Usage frequency of each heading term among identified RCTs with structured abstracts (n = 324). Table S4. Most frequent (>2%) patterns of heading term combinations among identified RCTs with structured abstracts (n = 324). Table S5. Characteristics of abstracts included for reporting quality assessment. Table S6. Association between quality of methodology reporting, structure formats and potential confounders - Univariable and multivariable linear regression derived coefficients (B) and 95% CIs, with overall quality score (OQS) as the dependent variable (n = 341). Table S7. Association between quality of methodology reporting, structure formats and potential confounders - Sensitivity analysis testing the definition for HS format - Univariable and multivariable linear regression derived coefficients (B) and 95% CIs, with overall quality score (OQS) as the dependent variable (n = 341). Table S8. Association between quality of methodology reporting, structure formats and potential confounders - Sensitivity analysis testing the definition used for HS format - Univariable and multivariable generalised estimation equation (GEE) derived coefficients (B) and 95% confidence intervals, with overall quality score (OQS) as the dependent variable and journal as the grouping factor (n = 341 from 33 journals). (DOCX 54 kb) [file 12874_2017_469_MOESM3_ESM.docx]

**Additional file 3**

**Additional file3: Table S1** Abstracts of RCTs published in top-50 journals in the ‘Medicine, General and Internal’ category during July-December 2015 - Characteristics by structure format

| **Characteristic** | **Unstructured (n=46)** | **IMRaD**  **(n=215)** | **HS**  **(n=109)** | **Overall**  **(n=370)** |
| --- | --- | --- | --- | --- |
| Journal type (%) |  |  |  |  |
| *General* | 45 (97.8) | 175 (81.4) | 65 (59.6) | 285 (77.0) |
| *Specialty* | 1 (2.2) | 40 (18.6) | 44 (40.4) | 85 (23.0) |
| Mean overall word count (SD) | 268.5 (67.4) | 340.6 (100.5) | 343.8 (70.5) | 332.6 (91.9) |
| Mean word count of methods section(s) (SD) | 79.8 (31.2) | 111.2 (49.8) | 129.6 (36.7) | 112.7 (46.6) |
| Median overall No. of paragraphs (IQR) | -- | 4.0 (4.0 to 5.0) | 8.0 (8.0 to 8.0) | 5.0 (4.0 to 8.0) **^a^** |
| Median No. of methods paragraphs (IQR) | -- | 1.0 (no variation) | 3.0 (3.0 to 4.0) | 1.0 (1.0 to 3.0) **^a^** |
| Median overall No. of heading terms (IQR) | -- | 4.0 (4.0 to 5.0) | 10.0 (8.0 to 11.0) | 5.0 (4.0 to 8.0) **^a^** |
| Median No. of heading terms regarding methods (IQR) | -- | 1.0 (1.0 to 1.0) | 5.0 (4.0 to 5.0) | 1.0 (1.0 to 4.0) **^a^** |
| Incorporated all 8 headings proposed by Haynes *et al* (%) **^b^** | 0 (0.0) | 0 (0.0) | 55 (50.5) | 55 (14.9) |

IMRaD: introduction, methods, results, and discussion format; HS: highly structured format;

SD: standard deviation; IQR: interquartile range (25th to 75th percentile).

**^a^** Calculation limited to structured abstracts (n=324).

**^b^** 8 headings: Objective, Design, Setting, Patients/Participants, Interventions, Main outcome measures, Results, Conclusions [9].

**Additional file3: Table S2.** Abstracts of RCTs published in top-50 journals in the ‘Medicine, General and Internal’ category during July-December 2015 - Characteristics by type of journal

| **Characteristic** | **General journals**  **(n=285)** | **Specialty journals**  **(n=85)** | **Overall**  **(n=370)** |
| --- | --- | --- | --- |
| Structure format (%) |  |  |  |
| *Unstructured* | 45 (15.8) | 1 (1.2) | 46 (12.4) |
| *IMRaD* | 175 (61.4) | 40 (47.1) | 215 (58.1) |
| *HS* | 65 (22.8) | 44 (51.8) | 109 (29.5) |
| Mean overall word count (SD) | 348.4 (94.7) | 279.3 (55.3) | 332.6 (91.9) |
| Mean word count of methods section(s) (SD) | 117.9 (49.1) | 95.2 (31.8) | 112.7 (46.6) |
| Median overall No. of paragraphs (IQR) **^a^** | 5.0 (4.0 to 7.0) | 5.0 (4.0 to 8.0) | 5.0 (4.0 to 8.0) |
| Median No. of methods paragraphs (IQR) **^a^** | 1.0 (1.0 to 3.0) | 2.0 (1.0 to 4.0) | 1.0 (1.0 to 3.0) |
| Median overall No. of heading terms (IQR) **^a^** | 5.0 (4.0 to 8.5) | 5.0 (4.0 to 8.0) | 5.0 (4.0 to 8.0) |
| Median No. of heading terms regarding methods (IQR) **^a^** | 1.0 (1.0 to 4.0) | 2.0 (1.0 to 4.5) | 1.0 (1.0 to 4.0) |
| Incorporated all 8 headings proposed by Haynes *et al* (%) **^b^** | 48 (16.8) | 7 (8.2) | 55 (14.9) |

IMRaD: introduction, methods, results, and discussion format; HS: highly structured format;

SD: standard deviation; IQR: interquartile range (25th to 75th percentile).

**^a^** Calculations limited to structured abstracts (n=324).

**^b^** 8 headings: Objective, Design, Setting, Patients/Participants, Interventions, Main outcome measures, Results, Conclusions [9].

**Additional file3: Table S3.** Usage frequency of each heading term among identified RCTs with structured abstracts (n=324)

| **Heading term** | **N (%)** | | |
| --- | --- | --- | --- |
|  | **IMRaD**  **(n=215)** | **HS**  **(n=109)** | **Overall**  **(n=324)** |
| Background | 165 (76.7) | 18 (16.5) | 183 (56.5) |
| Importance | 0 (0.0) | 45 (41.3) | 45 (13.9) |
| Introduction | 9 (4.2) | 7 (6.4) | 16 (4.9) |
| Context | 4 (1.9) | 0 (0.0) | 4 (1.2) |
| Objective(s) | 31 (14.4) | 99 (90.8) | 130 (40.1) |
| Aim | 2 (0.9) | 3 (2.8) | 5 (1.5) |
| Purpose | 7 (3.3) | 0 (0.0) | 7 (2.2) |
| Study question | 4 (1.9) | 0 (0.0) | 4 (1.2) |
| Method(s) | 212 (98.6) | 14 (12.8) | 226 (69.8) |
| Design | 7 (3.3) | 103 (94.5) | 110 (34.0) |
| Setting | 0 (0.0) | 89 (81.7) | 89 (27.5) |
| Participants | 0 (0.0) | 81 (74.3) | 81 (25.0) |
| Patients | 2 (0.9) | 8 (7.3) | 10 (3.1) |
| Subjects | 1 (0.5) | 4 (3.7) | 5 (1.5) |
| Population(s) | 0 (0.0) | 1 (0.9) | 1 (0.3) |
| Material(s) | 3 (1.4) | 0 (0.0) | 3 (0.9) |
| Intervention(s) | 0 (0.0) | 90 (82.6) | 90 (27.8) |
| Main outcome measure(s) | 0 (0.0) | 75 (68.8) | 75 (23.1) |
| Measures / measurements | 0 (0.0) | 14 (12.8) | 14 (4.3) |
| Outcome(s) | 0 (0.0) | 4 (3.7) | 4 (1.2) |
| Primary outcome | 0 (0.0) | 5 (4.6) | 5 (1.5) |
| Secondary outcome | 0 (0.0) | 2 (1.8) | 2 (0.6) |
| Exploratory outcomes | 0 (0.0) | 1 (0.9) | 1 (0.3) |
| Randomisation | 0 (0.0) | 4 (3.7) | 4 (1.2) |
| Blinding | 0 (0.0) | 1 (0.9) | 1 (0.3) |
| Analysis | 0 (0.0) | 1 (0.9) | 1 (0.3) |
| Results | 154 (71.6) | 109 (100.0) | 263 (81.2) |
| Findings | 57 (26.5) | 0 (0.0) | 57 (17.6) |
| Study Answer | 4 (1.9) | 0 (0.0) | 4 (1.2) |
| Conclusion(s) | 156 (72.6) | 109 (100.0) | 265 (81.8) |
| Discussion | 2 (0.9) | 0 (0.0) | 2 (0.6) |
| Interpretation | 54 (25.1) | 0 (0.0) | 54 (16.7) |
| Relevance | 0 (0.0) | 45 (41.3) | 45 (13.9) |
| Limitation | 4 (1.9) | 8 (7.3) | 12 (3.7) |
| What this study adds | 4 (1.9) | 0 (0.0) | 4 (1.2) |
| Funding (source) | 57 (26.5) | 7 (6.4) | 64 (19.8) |
| Trial registration (number) | 22 (10.2) | 73 (67.0) | 95 (29.3) |
| Competing interests | 4 (1.9) | 0 (0.0) | 4 (1.2) |
| Data sharing | 4 (1.9) | 0 (0.0) | 4 (1.2) |

IMRaD: introduction, methods, results, and discussion format; HS: highly structured format.

**Additional file 3: Table S4.** Most frequent (>2%) patterns of heading term combinations among identified RCTs with structured abstracts (n=324)

| **Patterns** | **N (%)**  **(n=324)** | **Structure format** |
| --- | --- | --- |
| 1. Background, Method(s), Results, Conclusion(s) | 94 (29.0) | IMRaD |
| 2. Background, Method(s), Findings, Interpretation, Funding | 53 (16.4) | IMRaD |
| 3. Importance, Objective(s), Design, Setting, Participants, Interventions, Main outcome measure(s), Results, Conclusions, Relevance, Trial registration | 45 (13.9) | HS |
| 4. Objective(s), Method(s), Results, Conclusion(s) | 15 (4.6) | IMRaD |
| 5. Objective(s), Design, Setting, Participants, Interventions, Main outcome measure(s), Results, Conclusion(s), Trial registration | 9 (2.8) | HS |
| 6. Background, Method(s), Results, Conclusion, Trial registration | 7 (2.2) | IMRaD |
| 7. Background, Objectives, Design, Setting, Patients, Interventions, Measure(s), Results, Limitation, Conclusion(s), Funding | 7 (2.2) | HS |
| Overall | 230 (71.1) |  |

IMRaD: introduction, methods, results, and discussion format; HS: highly structured format.

**Additional file 3: Table S5.** Characteristics of abstracts included for reporting quality assessment

| **Characteristic** | **IMRaD**  **(n=176)** | **HS**  **(n=165)** | **Overall**  **(n=341)** |
| --- | --- | --- | --- |
| Journal type (%) |  |  |  |
| *General* | 112 (63.6) | 60 (36.4) | 172 (50.4) |
| *Specialty* | 64 (36.4) | 105 (63.6) | 169 (49.6) |
| Continent of origin (%) |  |  |  |
| *Europe* | 75 (42.6) | 51 (30.9) | 126 (37.0) |
| *North America* | 49 (27.8) | 77 (46.7) | 126 (37.0) |
| *Asia* | 44 (25.0) | 11 (6.7) | 55 (16.1) |
| *Oceania* | 4 (2.3) | 22 (13.3) | 26 (7.6) |
| *Others* | 4 (2.3) | 4 (2.4) | 8 (2.3) |
| Publication year (%) |  |  |  |
| *2015* | 133 (75.6) | 126 (76.4) | 259 (76.0) |
| *2010 - 2014* | 43 (24.4) | 39 (23.6) | 82 (24.0) |
| No. of centres (%) |  |  |  |
| *Single centre* | 81 (46.0) | 55 (33.3) | 136 (39.9) |
| *Multi-centre* | 95 (54.0) | 110 (66.7) | 205 (60.1) |
| Funded (%) |  |  |  |
| *No* | 32 (18.2) | 11 (6.7) | 43 (12.6) |
| *Yes* | 144 (81.8) | 154 (93.3) | 298 (87.4) |
| Mean overall word count (SD) | 282.0 (70.9) | 310.6 (64.2) | 295.8 (69.1) |
| Mean word count of methods section(s) (SD) | 88.3 (33.1) | 116.4 (35.2) | 101.9 (36.9) |
| Median overall No. of paragraphs (IQR) | 4.0 (4.0 to 5.0) | 8.0 (6.0 to 9.0) | 5.0 (4.0 to 8.0) |
| Median No. of methods paragraphs (IQR) | 1.0 (1.0 to 1.0) | 4.0 (3.0 to 5.0) | 1.0 (1.0 to 3.0) |
| Median overall No. of heading terms (IQR) | 5.0 (4.0 to 5.0) | 9.0 (7.0 to 10.0) | 5.0 (5.0 to 9.0) |
| Median No. of heading terms regarding methods (IQR) | 1.0 (1.0 to 1.0) | 5.0 (4.0 to 5.0) | 2.0 (1.0 to 5.0) |
| Incorporated all 8 headings proposed by Haynes *et al* (%) **^a^** | 0 (0.0) | 69 (41.8) | 69 (20.2) |
| CONSORT for Abstracts endorsement (%) **^b^** |  |  |  |
| Not mentioned | 160 (90.9) | 135 (81.8) | 295 (86.5) |
| Recommended | 8 (4.5) | 16 (9.7) | 24 (7.0) |
| Required | 8 (4.5) | 14 (8.5) | 22 (6.5) |

IMRaD: introduction, methods, results, and discussion format; HS: highly structured format;

SD: standard deviation; IQR: interquartile range (25th to 75th percentile).

**^a^** 8 headings: Objective, Design, Setting, Patients/Participants, Interventions, Main outcome measures, Results, Conclusions [9].

**^b^** Editorial policy according to journals’ ‘instructions to authors’ (as of April 2016). CONSORT for Abstracts: the CONSORT (Consolidated Standards of Reporting Trials) extension guidelines for reporting of RCT abstracts [23].

**Additional file 3: Table S6.** Association between quality of methodology reporting, structure formats and potential confounders - Univariable and multivariable linear regression derived coefficients (*B*) and 95% CIs, with overall quality score (OQS) as the dependent variable (n=341)

|  | | **Univariable** | | | |  | **Multivariable** **^a^** | | | | |
| --- | --- | --- | --- | --- | --- | --- | --- | --- | --- | --- | --- |
| **Explanatory variable** | **Category/unit** | ***B*** | **95% CI** | **P value** | **Adjusted R^2^** |  | ***B*** | **95% CI** | **P value** | **Tolerance** | **VIF ^b^** |
| *Structure format* | IMRaD | Reference | | |  |  | Reference | | | |  |
|  | HS | 0.66 | (0.37, 0.94) | <0.001 | 0.053 |  | 0.69 | (0.40, 0.99) | **<0.001** | 0.818 | 1.223 |
| *Journal type* | General | Reference | | |  |  | Reference | | | |  |
|  | Specialty | -0.33 | (-0.62, -0.03) | 0.029 | 0.011 |  | -0.59 | (-0.88, -0.29) | **<0.001** | 0.811 | 1.233 |
| *Continent* |  |  |  | 0.022 | 0.022 |  |  |  |  |  |  |
|  | Europe | Reference | | |  |  | Reference | | | | |
|  | North America | -0.21 | (-0.55, 0.13) | 0.215 |  |  | -0.28 | (-0.61, 0.04) | 0.087 | 0.712 | 1.405 |
|  | Asia | -0.71 | (-1.15, -0.28) | 0.001 |  |  | -0.49 | (-0.90, -0.08) | **0.021** | 0.768 | 1.303 |
|  | Oceania | 0.09 | (-0.49, 0.67) | 0.763 |  |  | 0.07 | (-0.52, 0.67) | 0.811 | 0.704 | 1.421 |
|  | Others | -0.10 | (-1.09, 0.88) | 0.837 |  |  | -0.20 | (-1.10, 0.70) | 0.664 | 0.949 | 1.054 |
| *Publication year* | 1 year | 0.16 | (0.003, 0.317) | 0.046 | 0.012 |  | 0.30 | (0.14, 0.47) | **<0.001** | 0.783 | 1.277 |
| *No. of centres* | Single centre | Reference | | |  |  | Reference | | | |  |
|  | Multi-centre | 0.71 | (0.42, 1.00) | <0.001 | 0.061 |  | 0.48 | (0.19, 0.77) | **0.001** | 0.898 | 1.114 |
| *Funded* | No | Reference | | |  |  | Reference | | | |  |
|  | Yes | 0.98 | (0.55, 1.41) | <0.001 | 0.055 |  | 0.74 | (0.32, 1.17) | **0.001** | 0.865 | 1.156 |

IMRaD: introduction, methods, results, and discussion format; HS: highly structured format.

**^a^** For the final multivariable model, constant=-608.837, adjusted R^2^=0.193, P<0.001.

**^b^** VIF: variance inflation factor.

**Additional file 3: Table S7.** Association between quality of methodology reporting, structure formats and potential confounders - Sensitivity analysis testing the definition for HS format - Univariable and multivariable linear regression derived coefficients (*B*) and 95% CIs, with overall quality score (OQS) as the dependent variable (n=341)

|  | | **Univariable** | | | |  | **Multivariable ^a^** | | | | | |
| --- | --- | --- | --- | --- | --- | --- | --- | --- | --- | --- | --- | --- |
| **Explanatory variable** | **Category/unit** | ***B*** | **95% CI** | **P value** | **Adjusted R^2^** |  | ***B*** | **95% CI** | **P value** | | **Tolerance** | **VIF ^b^** |
| *Structure format* |  |  |  | <0.001 | 0.068 |  |  |  | |  |  |  |
|  | IMRaD | Reference | |  |  |  | Reference | | | |  |  |
|  | Other HS | 0.44 | (0.10, 0.77) | 0.011 |  |  | 0.62 | (0.26, 0.98) | | **0.001** | 0.678 | 1.475 |
|  | 8-heading | 0.96 | (0.59, 1.34) | <0.001 |  |  | 0.77 | (0.41, 1.14) | | **<0.001** | 0.826 | 1.211 |
| *Journal type* | General | Reference | | |  |  | Reference | | | | |  |
|  | Specialty | -0.33 | (-0.62, -0.03) | 0.029 | 0.011 |  | -0.55 | (-0.86, -0.23) | **0.001** | | 0.713 | 1.402 |
| *Continent* |  |  |  | 0.022 | 0.022 |  |  |  |  | |  |  |
|  | Europe | Reference | | |  |  | Reference | | | | | |
|  | North America | -0.21 | (-0.55, 0.13) | 0.215 |  |  | -0.28 | (-0.60, 0.05) | 0.098 | | 0.708 | 1.412 |
|  | Asia | -0.71 | (-1.15, -0.28) | 0.001 |  |  | -0.47 | (-0.89, -0.06) | **0.026** | | 0.760 | 1.316 |
|  | Oceania | 0.09 | (-0.49, 0.67) | 0.763 |  |  | 0.08 | (-0.52, 0.67) | 0.803 | | 0.704 | 1.421 |
|  | Others | -0.10 | (-1.09, 0.88) | 0.837 |  |  | -0.21 | (-1.11, 0.69) | 0.647 | | 0.948 | 1.055 |
| *Publication year* | 1 year | 0.16 | (0.003, 0.317) | 0.046 | 0.012 |  | 0.30 | (0.14, 0.46) | **<0.001** | | 0.776 | 1.289 |
| *No. of centres* | Single centre | Reference | | |  |  | Reference | | | | |  |
|  | Multi-centre | 0.71 | (0.42, 1.00) | <0.001 | 0.061 |  | 0.49 | (0.20, 0.77) | **0.001** | | 0.895 | 1.117 |
| *Funded* | No | Reference | | |  |  | Reference | | | | |  |
|  | Yes | 0.98 | (0.55, 1.41) | <0.001 | 0.055 |  | 0.74 | (0.31, 1.17) | **0.001** | | 0.862 | 1.160 |

IMRaD: introduction, methods, results, and discussion format; HS: highly structured format; 8 headings: Objective, Design, Setting, Patients/Participants, Interventions, Main outcome measures, Results, Conclusions [9].

**^a^** For the final multivariable model, constant=-597.664, adjusted R^2^=0.192, P<0.001.

**^b^** VIF: variance inflation factor.

**Additional file 3: Table S8.** Association between quality of methodology reporting, structure formats and potential confounders - Sensitivity analysis testing the definition used for HS format - Univariable and multivariable generalised estimation equation (GEE) derived coefficients (*B*) and 95% confidence intervals, with overall quality score (OQS) as the dependent variable and journal as the grouping factor (n=341 from 33 journals)

|  | | **Univariable** | | | |  | **Multivariable ^b^** | | |
| --- | --- | --- | --- | --- | --- | --- | --- | --- | --- |
| **Explanatory variables** | **Category/unit** | ***B*** | **95% CI** | **P value** | **QICC ^a^** |  | ***B*** | **95% CI** | **P value** |
| *Structure format* |  |  |  | 0.005 | 612.9 |  |  | |  |
|  | IMRaD | Reference | | |  |  | Reference | |  |
|  | Other HS | 0.52 | (0.01, 1.02) | 0.045 |  |  | 0.39 | (-0.09, 0.88) | 0.108 |
|  | 8-heading | 0.84 | (0.32, 1.36) | 0.002 |  |  | 0.75 | (0.24, 1.26) | **0.004** |
| *Journal type* | General | Reference | | |  |  |  | |  |
|  | Specialty | -0.30 | (-0.86, 0.25) | 0.285 | 650.3 |  |  |  |  |
| *Continent* |  |  |  | 0.838 | 651.0 |  |  |  |  |
|  | Europe | Reference | | |  |  |  | | |
|  | North America | -0.16 | (-0.55, 0.22) | 0.408 |  |  |  |  |  |
|  | Asia | -0.28 | (-0.86, 0.31) | 0.353 |  |  |  |  |  |
|  | Oceania | 0.20 | (-0.63, 1.03) | 0.638 |  |  |  |  |  |
|  | Others | -0.23 | (-1.29, 0.84) | 0.677 |  |  |  |  |  |
| *Publication year* | 1 year | 0.09 | (-0.06, 0.25) | 0.224 | 652.7 |  |  |  |  |
| *No. of centres* | Single centre | Reference | | |  |  | Reference | |  |
|  | Multi-centre | 0.42 | (0.16, 0.68) | 0.002 | 623.9 |  | 0.37 | (0.10, 0.64) | **0.008** |
| *Funded* | No | Reference | | |  |  | Reference | |  |
|  | Yes | 0.69 | (0.39, 0.99) | <0.001 | 625.5 |  | 0.58 | (0.25, 0.91) | **0.001** |

IMRaD: introduction, methods, results, and discussion format; HS: highly structured format; 8-headings: Objective, Design, Setting, Patients/Participants, Interventions, Main outcome measures, Results, Conclusions [9].

**^a^** QICC, Corrected quasi likelihood under independence model criterion.

**^b^** For the final multivariable model, intercept=3.424, QICC=571.9.
